# Short-Term Surveillance of Cytokines and C-Reactive Protein Cannot Predict Efficacy of Fecal Microbiota Transplantation for Ulcerative Colitis

**DOI:** 10.1371/journal.pone.0158227

**Published:** 2016-06-27

**Authors:** Ting Zhang, Bota Cui, Pan Li, Zhi He, Chuyan Long, Lu Wei, Zhaoyuan Peng, Guozhong Ji, Faming Zhang

**Affiliations:** 1 Medical Center for Digestive Diseases, the Second Affiliated Hospital of Nanjing Medical University, Nanjing, Jiangsu Province, China; 2 Key Lab of Holistic Integrative Enterology, Nanjing Medical University, Nanjing, Jiangsu Province, China; University Hospital Llandough, UNITED KINGDOM

## Abstract

**Objective:**

There were no reports on predicting long-term efficacy of fecal microbiota transplantation (FMT) for ulcerative colitis (UC). This study aimed to detect short-term changes of cytokines and C-reactive protein (CRP) in patients with UC undergoing FMT, and to evaluate the predictive value of CRP and cytokines for the long-term efficacy of FMT.

**Methods:**

Nineteen patients with moderate to severe UC (Mayo score ≥ 6) were treated with single fresh FMT through mid-gut. Serum samples were collected before and three days post-FMT. Clinical responses were evaluated by a minimum follow-up of three months. Patients with clinical improvement and remission at the assessment point of three-month were included as response group, while patients without clinical improvement or remission were included as non-response group. Serum concentrations of cytokines (IL-1β, IL-2, IL-4, IL-6, IL-10, IL-11, IL-17A, IFN-γ, TNF, TNFR-1, TNFR-2, MCP-1, G-CSF, GM-CSF) and CRP were assayed to predict the clinical response of FMT.

**Results:**

In total, 10.5% (2/19) of patients achieved clinical remission and 47.4% (9/19) achieved clinical improvement (Response group, including clinical remission and clinical improvement), 42.1% (8/19) failed to benefit from FMT (Non-response group). In both Response group and Non-response group, the level of CRP at three days after FMT didn’t show significant decrease compared with that before FMT (p>0.05). However, in Response group, CRP level at three months after FMT decreased significantly than that before FMT (p<0.05). Compared with healthy controls (n = 9), patients with UC showed a higher baseline level of serum IL-6, TNFR-2 and G-CSF, and a lower level of IL-2 and IL-4 (p<0.05). In both Response group and Non-response group, none of the eleven detectable cytokines showed a significant difference between the value at three days after FMT and that before FMT (p>0.05).

**Conclusions:**

Patients with moderate to severe UC presented a complex disorder of cytokines. However, the efficacy of FMT for UC might not be predicted by the short-term surveillance of cytokines and CRP.

## Introduction

Ulcerative colitis (UC) is a chronic inflammatory bowel disease characterized by remitting and relapsing inflammation and epithelial injury[1,2]. Dysbiosis of the intestinal microbiota has been implicated in the etiology of this disease[3]. Fecal microbiota transplantation (FMT), a concept for the reconstruction of the intestinal homeostasis, originated in China in the fourth century[4]. Previous reports have shown the potential therapeutic role of FMT for the treatment of inflammatory bowel disease (IBD)[5–13]. In our previous study[14], the clinical response was better at one month after FMT, which might indicate slow or delayed response to FMT. Angelberger et al[15] also reported one patient with UC achieved positive clinical response at 12 weeks after FMT. Since FMT might bring long-term efficacy for the patients, we intended to find an immediate blood marker or markers for indicating the long-term outcome of FMT. C-reactive protein (CRP) was conventionally used to reflect the activity of IBD; however, both recent studies[15] and our group[14] observed that the temporary increase of CRP occurred after FMT in some cases, regardless whether they benefited from FMT or not. Cytokines have been proven to play a crucial role in controlling intestinal inflammation and associated clinical symptoms[16]. Disorders of cytokines on the circulating levels have been reported in patients with IBD[17–20]. The normal production of cytokines, including interleukin (IL)-1β[21], IL-2[22], IL-6[23], tumor necrosis factor (TNF)[16,24], interferon (IFN)-γ[25], IL-17[26], have been shown to be different from the normal controls. IBD is also related to gut dysbiosis, which plays a key role in regulating the host immune system[27–29]. However, it remains unknown whether the restoring of gut microbiota would affect the production of cytokines in IBD.

To determine whether there are markers which can be used to predict the long-term outcome of FMT, the changes of 14 cytokines in patients with UC at three days after FMT were evaluated to clarify the correlation between short-term changing of cytokines and long-term clinical efficacy of FMT.

## Materials and Methods

### Recruitment of patients

A prospective study as a part of clinical trial (NCT 01790061) was carried out by the Medical Center for Digestive Diseases at the Second Affiliated Hospital of Nanjing Medical University, Nanjing, China, from June 2013 to March 2015. This study was reviewed and approved by the Second Affiliated Hospital of Nanjing Medical University Institutional Review Board. All eligible subjects provided written informed consent prior to participation in the study. The diagnosis of UC consists of typical clinical, endoscopic, and histological criteria. All included patients aged 18–70 years old, had moderate to severe extensive UC (total Mayo score≥6)[30]. All participants were followed up more than three months. Exclusion criteria were: subjects who had an acute or chronic infectious disease; any clinically significant disorder e.g. cancer; subjects who were on any medication with a known effect on immunological factors, such as corticosteroids or immunomodulators; cases with incomplete samples or data.

### Efficacy assessment of FMT

The efficacy of FMT was evaluated by patients’ clinical symptoms involving abdominal pain, stool frequency, rectal bleeding and laboratory tests at different observation time points including three days, one week, one month and three months after FMT. To avoid frequent endoscopic examinations, the scores of endoscopic examination at one week and one month were supposed to be equal to that before FMT. Clinical remission is defined as total Mayo score ≤ 2 points, with no individual subscore ≥1. Clinical improvement is defined as a decrease from baseline in the total Mayo score of at least 3 points or at least 30%, along with a reduction in the rectal bleeding subscore (RBS) of at least 1 point or an absolute RBS of ≤ 1. All patients who achieved clinical remission were also included in the analysis of clinical improvement. For analysis, at the surveillance point of three-month, patients with clinical improvement and remission were included as response group, while patients without clinical improvement or remission were included as non-response group.

### Procedure of FMT

Firstly, patients were given metoclopramide 10 mg by intramuscular injection and esomeprazole magnesium 40 mg intravenously one hour before FMT. Fresh fecal material from selected donors was purified through our laboratory process based on a newly developed automatic purification system (GenFMTer, FMT Medical, Nanjing, China) as previously reported[14] since April 2014. Then the purified microbiota suspension was transplanted into the mid-gut of patients through an endoscopic infusion tube inserted into the gastroscope channel under anesthesia. Importantly, complete suction of stomach fluid must be performed before the endoscope was inserted into duodenum for infusion of microbiota suspension. Taken together, the entire procedure from the microbiota preparation to infusion should be completed within one hour.

### Determination of the cytokine levels

To evaluate the temporary changing of serum cytokines, blood samples were collected from UC patients (n = 19) on admission day and three days post-FMT. Because patients’ response to FMT can generally be confirmed by clinical parameters within three days after FMT according to our clinical experience, then the timepoint of three days was selected for assessing short-term response of cytokines. In addition, blood samples were collected from 9 healthy volunteers as control group. The control group was matched with the UC group on baseline characteristics, including age, gender, ethnicity, Body Mass Index (BMI). All subjects underwent laboratory evaluation including blood test (complete blood count), CRP, erythrocyte sedimentation rate (ESR) and biochemical tests.

The 14 cytokines known to or hypothesized to be involved in inflammatory processes and that were investigated were interleukin IL-1β, IL-2, IL-4, IL-6, IL-10, IL-11, IL-17A, IFN-γ, TNF, TNF receptor type 1 (TNFR-1), TNFR-2, monocyte chemoattractant protein (MCP)-1, granulocyte-colonystimulating factor (G-CSF) and granulocyte-macrophage colony-stimulating factor (GM-CSF). Fasting blood tests were sampled at seven or eight in the morning before FMT and on day 3 after FMT. Blood samples were first centrifuged at 2500 rpm for 20 min and the serum samples were then stored at −80°C until analysis. The circulating levels of fourteen cytokines were analyzed using BD Cytometric Bead Array Flex Set System kits (BD Biosciences, USA) according to the manufacturer’s instructions.

### Statistical analysis

Data were performed using SPSS (Chicago, IL, USA) or GraphPad (La Jolla, CA, USA). When the normality of the distribution of variables was acceptable, the independent sample t-test and paired student’s t test were used. When the normality of the distribution of variables was not acceptable, the Mann–Whitney U test and Wilcoxon signed-rank test were used to analyze differences between groups. Comparisons of categorical variables between groups were performed using a Fisher’s exact test. A value of p < 0.05 (two-tailed) was considered to indicate significance.

## Results

### Patient characteristics

A total of 19 patients (Table 1) with moderate-severe UC (Mayo score ≥ 6) were enrolled. In patients, the mean age was 39.2 year-old (range 19–60 years), and the mean disease duration was 8.0 years (range 1–21 years), 57.9% (11/19) had severe (Mayo score ≥ 11) disease and 42.1% (8/19) had moderate UC. In the control group, the mean age was 38.1 year-old, and 44.4% (4/9) were male.

**Table 1:** The characteristics of the included patients.

	Items	Results
Patient	Total number	19
	Age (years), $\bar{x}$ ± SD (range)	39.2 ± 14.1 (19–60)
	Sex, male% (n)	36.8 (7)
	Disease duration (years, $\bar{x}$ ± SD)	8.0 ± 5.8
	Mayo scores ($\bar{x}$ ± SD)	10.5 ± 1.7
	Smoking, yes % (n)	10.5 (2)
	With history of steroid, yes % (n)	21.1 (4)
	With history of immunomodulator, yes %(n)	21.1 (4)
	CRP before FMT (mg/l, $\bar{x}$ ± SD)	16.42 ± 10.53
	ESR before FMT (mm, $\bar{x}$ ± SD)	28.53 ± 18.04
	WBC before FMT (*10^9^/mm^3^, $\bar{x}$ ± SD)	7.50± 2.33
	Hb (g/l, $\bar{x}$ ± SD)	121.05 ± 21.34
	ALB (g/l, $\bar{x}$ ± SD)	41.63 ± 10.69
	IgG (g/l, $\bar{x}$ ± SD)	14.29 ± 3.09
	IgA(g/l, $\bar{x}$ ± SD)	2.23 ± 0.75
	IgM (g/l, $\bar{x}$ ± SD)	1.24 ± 0.39

Note: SD, (Standard Deviation); CRP, (C-reactive protein); ESR (erythrocyte sedimentation rate).

### Response to FMT

UC-related abdominal pain, stool frequency, bloody purulent stool, ESR, CRP and other parameters were assessed for all patients after FMT. According to the criteria of Mayo score, 31.6% (6/19) of patients benefited from the FMT at our first surveillance point of three-day. While at the three-month observing point, 57.9% (11/19) of patients achieved clinical response (named as response group), and 42.1% (8/19) of patients failed to benefit from the FMT (named as non-response group). Among the response group, 10.5% (2/19) met the criteria of clinical remission, and 47.4% (9/19) achieved clinical improvement. In addition, four patients in response group, who failed to achieve clinical improvement at three days, were observed to successfully achieve clinical efficacy three months post-FMT. In non-response group, two patients presented transient clinical improvement at three days, but did not achieve clinical improvement at three months.

In addition, we tested the correlation between the potential impact factors (including patients’ age, disease duration, smoking history etc.) and patients’ clinical response at three months post-FMT. As shown in Table 2, there was no significant difference among all investigated factors between two groups.

**Table 2:** Patients’ characteristics and clinical response.

Characteristics	Response(n = 11)	Non-Response(n = 8)	P
Age (years)	42.91 ± 15.27	34.00 ± 11.22	0.181
Sex (male/female)	3/8	4/4	0.377
Disease duration (years, m ± SD)	7.27 ± 5.57	8.88 ± 6.33	0.566
Age at onset (years, m ± SD)	35.64 ± 12.30	25.13 ± 10.84	0.070
Smoking (no/yes)	10/1	7/1	0.678
With history of steroid (no/yes)	9/2	6/2	0.574

Note: SD, (Standard Deviation).

### Safety of FMT

No severe adverse events were observed during and after FMT procedure, as well as during the three months’ follow-up. 36.8% of patients (7/19) had a transient increased diarrhea frequency within 24h after FMT, and mainly occurred within three hours after FMT. One patient occurred mild skin pruritus six hours after FMT, and two patients presented with borborygmus. All symptoms resolved without any medical intervention. Except the above events, there was no more adverse event during the follow-up of more than three months.

### Changing levels of CRP and clinical response

**Fig 1:**
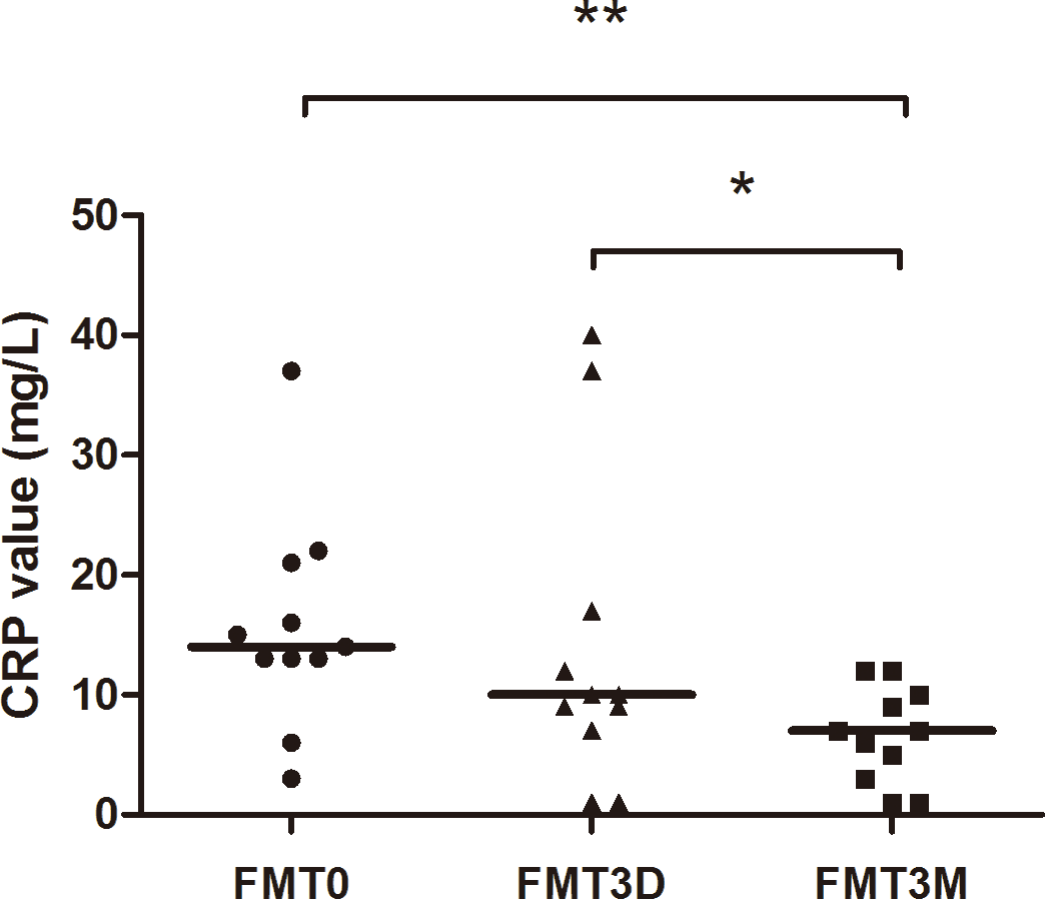
Surveillance of CRP in response group (n = 11). There is no statistical difference on CRP levels in pre-FMT and three days post-FMT. Compared with the value pre-FMT and three days post-FMT, the level of CRP decreased three months post-FMT, ** p<0.01 and * p<0.05, respectively. FMT0: 0 day pre-FMT, FMT3D: three days post-FMT, FMT3M: three months post-FMT.

The CRP levels at three days post-FMT didn’t show a significant difference compared with that before FMT in both response group and non-response group (p>0.05). Two patients who showed an increased level of CRP at three days still maintained clinical improvement at the three-month follow-up point. However, the patients who achieved clinical improvement and clinical remission at three months post-FMT had a significantly lower CRP level than that before FMT (6.64±3.93mg/l vs. 15.73±8.96 mg/l, p = 0.003) (Fig 1), while CRP in non-response group didn’t show a similar trend (p>0.05).

### Changes of cytokines after FMT

The baseline values of cytokines in UC patients before FMT were evaluated and then compared with healthy controls. As shown in Table 3, patients with UC presented a distinct cytokine profile compared with the healthy controls. Among the 14 cytokines, three cytokines (IL-1β, IL-11 and GM-CSF) were not detectable in both groups. The levels of serum IL-6, TNFR-2 and G-CSF in UC patients were significantly higher than that in the healthy persons (p<0.05), while IL-2 and IL-4 were significantly decreased (p<0.05). Six cytokines (IL-10, IL-17A, IFN-γ, TNF, TNFR-1 and MCP-1) did not show a significant difference between the UC patients and healthy persons.

**Table 3:** Concentrations of cytokines in UC and the healthy controls.

cytokine	Controls (n = 9)	UC (n = 19)	P
IL-1β	nd	nd	-
IL-2	3.66 (1.91–5.80)	1.25 (nd-4.48)*	0.003
IL-4	3.57 (2.76–5.18)	2.12 (nd-2.87)*	0.020
IL-6	4.89 (3.415–5.56)	9.185 (5.375–18.148)*	0.002
IL-10	3.55 (0.88–3.785)	1.92 (nd-3.388)	0.275
IL-11	nd	nd	-
IL-17A	19.34 (14.62–24.16)	20.41 (11.97–30.73)	0.594
IFN-γ	4.05 (2.67–4.53)	2.46 (nd-3.953)	0.072
TNF	2.98 (nd-3.55)	3.47 (nd-4.78)	0.236
TNFR-1	34.68 (9.925–73.865)	44.22 (14.268–144.578)	0.640
TNFR-2	634.55 (365.71–1005.33)	1685.52 (1017.775–2223.61)*	0.004
MCP-1	10.73 (4.805–17.170)	19.635 (11.453–30.443)	0.161
G-CSF	nd	0.675 (nd-2.628)*	0.007
GM-CSF	nd	nd	-

Note: Values are expressed as the median (interquartile range) in pg/ml.

* p<0.05, vs. control group (Mann-Whitney test with Bonferroni correction for multiple comparisons).

nd: non-detectable, IBD: inflammatory bowel disease, IL: interleukin, IFN: inter feron, TNF: tumor necrosis factor, TNFR-1: tumor necrosis factor receptor-1, TNFR-2: tumor necrosis factor receptor-1, MCP: monocyte chemoattractant protein, G-CSF: granulocyte-colony stimulating factor, GM-CSF: granulocyte-macrophage colony-stimulating factor.

**Fig 2:**
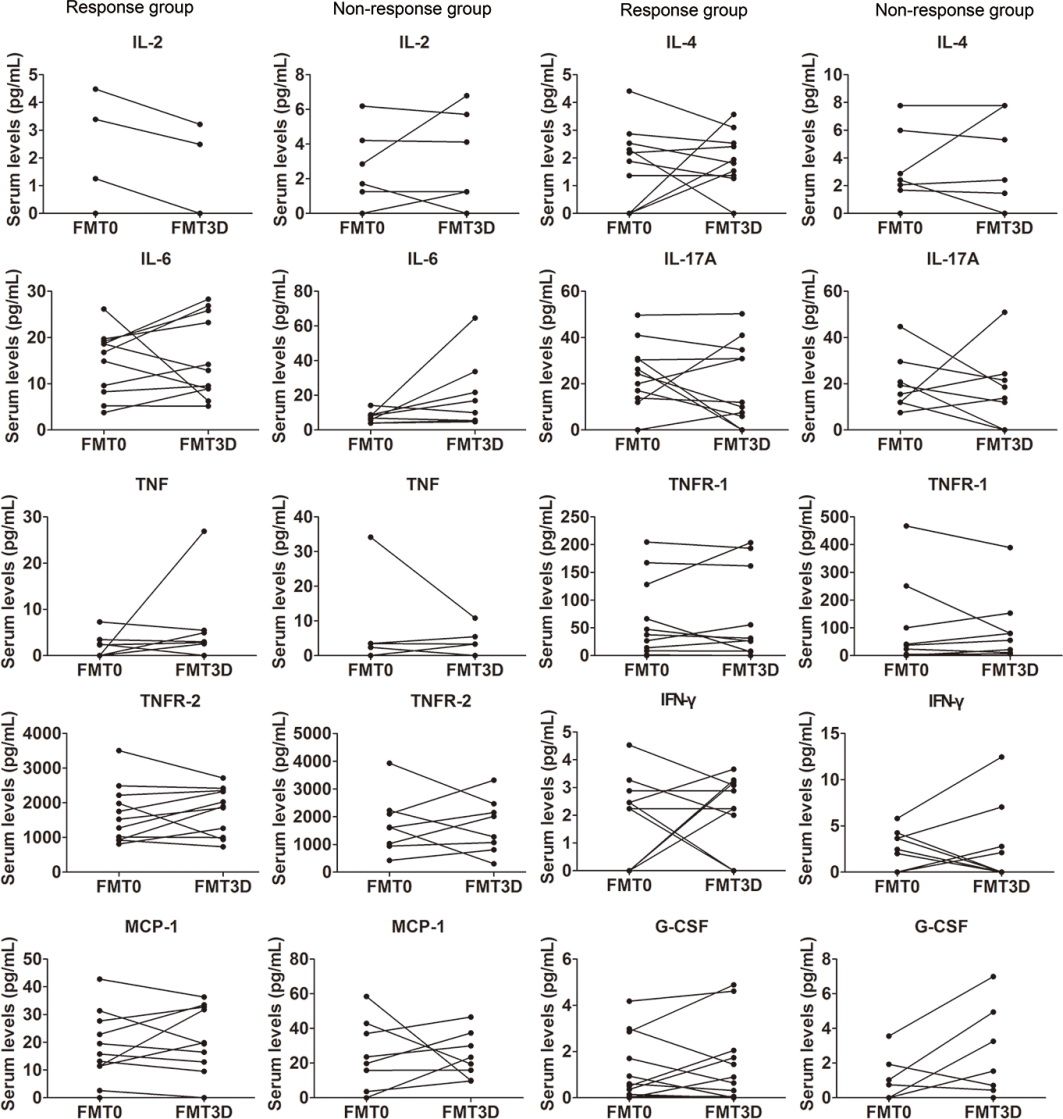
Changes of cytokines before and after FMT in patients with UC. The serum concentrations of cytokines (IL-1β, IL-2, IL-4, IL-6, IL-10, IL-11, IL-17A, IFN-γ, TNF, TNFR-1, TNFR-2, MCP-1, G-CSF, GM-CSF) between pre-FMT group and three days post-FMT group were not significantly different (p>0.05).

Concentrations of cytokines at three days after FMT were analyzed and compared with the original values before FMT in patients with UC. All of the eleven detectable cytokines didn’t show a significant difference between the values at three days after FMT and the values before FMT (p>0.05), regardless whether the patients benefited from FMT or not (Fig 2).

## Discussion

In the present study, 10.5% (2/19) of patients achieved clinical improvement and 47.4% (9/19) achieved clinical remission at three months after FMT. These results indicated that the therapeutic role of FMT for UC was consistent with Moayyedi’s study[6]. However, the efficacy of FMT for UC was still in debate. Rossen et al[13] and Kump et al[31] reported no benefit was achieved from FMT in patients with UC. The controversy in the efficacy of FMT might be explained by the difference in the methodology of FMT[32].

FMT had been reported to ameliorate the abdominal pain and reduce the frequency of diarrhea immediately in the patients with Crohn’s diseases in our previous study[14]. However, some studies[14,15] reported that a delayed positive clinical response to FMT was observed in IBD. In this study, we observed that four patients with UC, who failed to achieve clinical improvement at three days, successfully achieved clinical efficacy at three months post-FMT, ultimately. Two patients achieved transient clinical response at three days post-FMT, but relapsed soon. These results implied that both immediate and delayed clinical response should be highlighted during assessment of FMT efficacy.

Some quantifiable laboratory assessments including CRP, ESR, leukocytes, platelets and lymphocytes subset analysis were used to evaluate the disease activity of IBD in our previous study and some other studies[7,33,34]. These available serum lab assays may be helpful to answer the question whether the patient is getting better with the treatment of FMT. This is also the reason why these markers were included in this study.

CRP is a sensitive and reliable index of inflammatory process. The level of CRP increases in patients with trauma, inflammation and infection[35]. Determination of CRP is very important in diagnosis, treatment and monitoring of inflammatory conditions, because an elevated CRP level is always associated with pathological changes. In the patients who benefited from FMT, the level of CRP did not decrease three days after FMT compared with that before FMT, indicating that the three days’ value of CRP might not be able to predict the immediate clinical efficacy. However, in the response group, CRP decreased significantly at the assessment point of three months after FMT.

Among the cytokines investigated in the present study, IL-6, TNFR-2 and G-CSF were shown to be significantly upregulated in UC patients, while IL-2 and IL-4 were significantly downregulated, as compared with the controls. IL-6, in particular, is known to have a strong proinflammatory reaction[36], certain other cytokines, such as IL-4, may play a part in decreasing the inflammatory activity[37].

TNF is a proinflammatory cytokine, which has been reported to play a critical role in promoting chronic inflammation due to its pleiotropic functions[38–40]. Furthermore, anti-TNF monoclonal antibodies have been proved to be effective in the treatment of UC and CD[41]. TNF-α, as a potent cytokine promoting IBD, showed a trend of rise in serum of UC patients. However, it didn’t show a significant difference between UC patients and the controls in this study. The similar results were reported in pediatric patients with inflammatory bowel disease[42]. These results were completely unexpected, but may contribute to explain why certain IBD patient failed to benefit from anti-TNF therapy.

Levels of TNFR1 and TNFR2 were also tested in this study. TNF may exert various proinflammatory functions in colitis by binding to its receptors TNFR1 and TNFR2 followed by the intracellular activation of the transcription factor nuclear factor-κB (NF-κB). Recent clinical and experimental studies have shown that membrane-bound TNF, rather than soluble TNF, plays an important role in driving intestinal inflammation. Consistent with this, neutralization of membrane-bound TNF has been shown to induce T cell apoptosis and was effective in suppressing experimental colitis in mice, whereas activation of TNFR2 (which is induced by membrane-bound but not soluble TNF) on T cells was found to aggravate colitis activity[43,44]. It may account for the fact that serum concentrations of TNFR-2 were significantly elevated in IBD patients.

IL-2 is a proinflammatory factor mainly produced by Th1 cells, which could activate NK cells and macrophages and strengthen the sterilization ability of immunocyte to pathogens. It’s unexpected that the concentration of IL-2, which has been previously reported to be upregulated at the mucosal level, was significantly downregulated particularly considering that in IBD patients. In accordance with our results, recent studies have found that expression of IL-2 is downregulated at the level of the inflamed mucosa in murine models[16], indicating that there may be a negative correlation between the level of IL-2 and the severity of UC. More studies are needed to further verify our hypothesis.

Therefore, the results of the present study demonstrated that the characteristics of immune response in IBD patients might be more complicated than originally considered, and may be associated with certain aspect of immunodeficiency. It’s important to evaluate and figure out the precise balance between proinflammatory and anti-inflammatory cytokines during the occurrence and development of IBD.

In non-response group, all of the eleven detectable cytokines didn’t show a statistic difference at three days after FMT compared with the values before FMT. Surprisingly, there was also no significant integral difference on response group in pre-therapy and post-treatment. Even a number of these molecules, including IL-2, IL-4, IL-6, TNFR-2 and G-CSF, which have been previously reported to be statistically significant when compared with the normal controls, didn’t show a statistical difference before and after treatment in UC patients. These results indicated that clinical efficacy in patients with UC couldn’t be predicted by the cytokine changes at a short-term surveillance point of three days post-FMT. In another word, these results indicated that detection of cytokines is possibly not a strong evidence for the evaluation of curative effect in patients with UC.

There are some limitations in the present study. First, the small sample size may be responsible for some surprising results and a larger sample study is needed to conduct further analysis. Then CRP data should have been shown for both response and non-response groups. However, because of the consideration on the cost of tests and ethical reasons, we didn’t do the CRP tests since we confirmed some patients who had no response to FMT according to clinical assessments. In addition, the serum concentrations of cytokines three months after FMT were not tested in this study. Further studies are necessary to improve the understanding of IBD etiology and clarify whether there are differences in serum levels of cytokines between three days and three months after FMT.

In conclusion, FMT has showed its therapeutic role in UC. Patients with UC presented a complex disorder of cytokines. However, the efficacy of FMT for UC cannot be predicted by the short-term surveillance of cytokines and CRP.

### Ethics Statement

This study was reviewed and approved by the Second Affiliated Hospital of Nanjing Medical University Institutional Review Board. All eligible subjects provided written informed consent prior to participation in the study.

## Supporting Information

S1 File Cytokine changes between pre-FMT and post-FMT in patients with UC. (XLS)
